# Complete chloroplast genome sequences of *Lagotis yunnanensis* (Scrophulariaceae): an Endangered species endemic to the Hengduan Mountains region

**DOI:** 10.1080/23802359.2020.1717394

**Published:** 2020-01-27

**Authors:** Jing-Ping Cheng, Ying-Min Zhang, Zi-Gang Qian, Guo-Dong Li

**Affiliations:** Faculty of Traditional Chinese Pharmacy, Yunnan University of Chinese Medicine, Kunming, China

**Keywords:** *Lagotis yunnanensis*, chloroplast genome, phylogenomic analysis

## Abstract

*Lagotis yunnanensis* is a perennial plant in the Scrophulariaceae family with a high value of medicinal in Tibetan medicine. In this study, we assembled and characterized the complete chloroplast genome of *L. yunnanensis* as a resource for future studies on this species. The chloroplast genome was 152,789 bp in size, with a large single-copy (LSC) region of 83,642 bp, a small single-copy (SSC) region of 17,795 bp, separated by two inverted repeat (IR) regions of 25,676 bp each. A total of 131 genes were predicted. Phylogenetic analysis showed a close relationship between *L. yunnanensis* and *Veronicastrum sibiricum* with 100% bootstrap value.

*Lagotis yunnanensis* W. W. Smith is a perennial plant of the genus *Lagotis* that is distributed in alpine grasslands at high altitudes of 3350–4700 m in northwestern Yunnan, Tibet, and northwestern Sichuan (Editorial Committee of Flora of China 1979). It has been used in Tibetan folk medicine for the treatment of fever, hypertension, acute and chronic hepatitis (Zhu et al. 2017). The chemical composition of the plant has been reported like flavonoids, Iridoid glycosides, etc (Yang et al. 2005, 2007). Recently, *Lagotis* gained more academic attention owning to effective components and pharmacological activity with a long history as an ethnomedicine (Zhang 2016); however, until now, little is known about in its molecular biology. Here, we reported the complete chloroplast genome sequence of *L. yunnanensis* and revealed its phylogenetic relationships with other species, which can provide the basis for the development and utilization of *L. yunnanensis* and its related genus.

The individual used for sequencing was sampled from Jianchuan (26°73′N, 99°43′E), Yunnan province in China. The voucher specimen was deposited at the Herbarium of Yunnan University of Chinese Medicine with accession number 5329311213. Total DNA was extracted from fresh leaves and used for library construction. The total genomic DNA was extracted using plant DNA (Bioteke Corporation, China) and sequencing was performed on an Illumina HiSeq 2500 platform (Illumina Inc., SanDiego, CA). The filtered reads were assembled using NOVOPlasty (Dierckxsens et al. 2017) with complete genome of its close relative *Veronicastrum sibiricum* as the reference. The assembled chloroplast genome was annotated with the online annotation tool GeSeq (Tillich et al. 2017), and the annotation was corrected using Geneious R11 11.1.5 (Biomatters Ltd., Auckland, New Zealand).

The complete chloroplast genome of *L. yunnanensis* (GenBank Accession No. MN752238) was 152,789 bp in length and a typical circular structure comprising a pair of inverted repeats (IR) of 25,676 bp divided by a large single copy (LSC) region of 83,642 bp and a small single copy (SSC) region of 17,795 bp. The whole genome contained 131 genes, including 86 protein-coding genes, 8 ribosomal RNA genes, and 37 tRNA genes. The general GC content was 38.4% in the whole sequence, while the corresponding values of the LSC, SSC, and IR regions are 36.6%, 32.8%, and 43.3%, respectively.

To determine the phylogenetic position of *L. yunnanensis*, a phylogenetic analysis was performed using the complete chloroplast genome of *L. yunnanensis* with those from seven Plantaginaceae species (including four from the genus *Plantago*, three from other genus) and Scrophulariaceae species (including four from the genus *Scrophularia*). *Orobanche* and *Pedicularis* (Orobanchaceae) were used as outgroups. All of the plastomes were aligned using MAFFT v.7 (Katoh and Standley 2013), and the RAxML (Stamatakis 2014) inference was performed by using the GTR model with support for branches evaluated by 1000 bootstrap replicates. The phylogenetic tree showed that *L. yunnanensis* is sister to *Veronicastrum sibiricum* (Figure 1). Our data can provide a useful resource for the conservation genetics of *L. yunnanensis* as well as for the phylogenetic studies of Scrophulariaceae.

**Figure 1.**
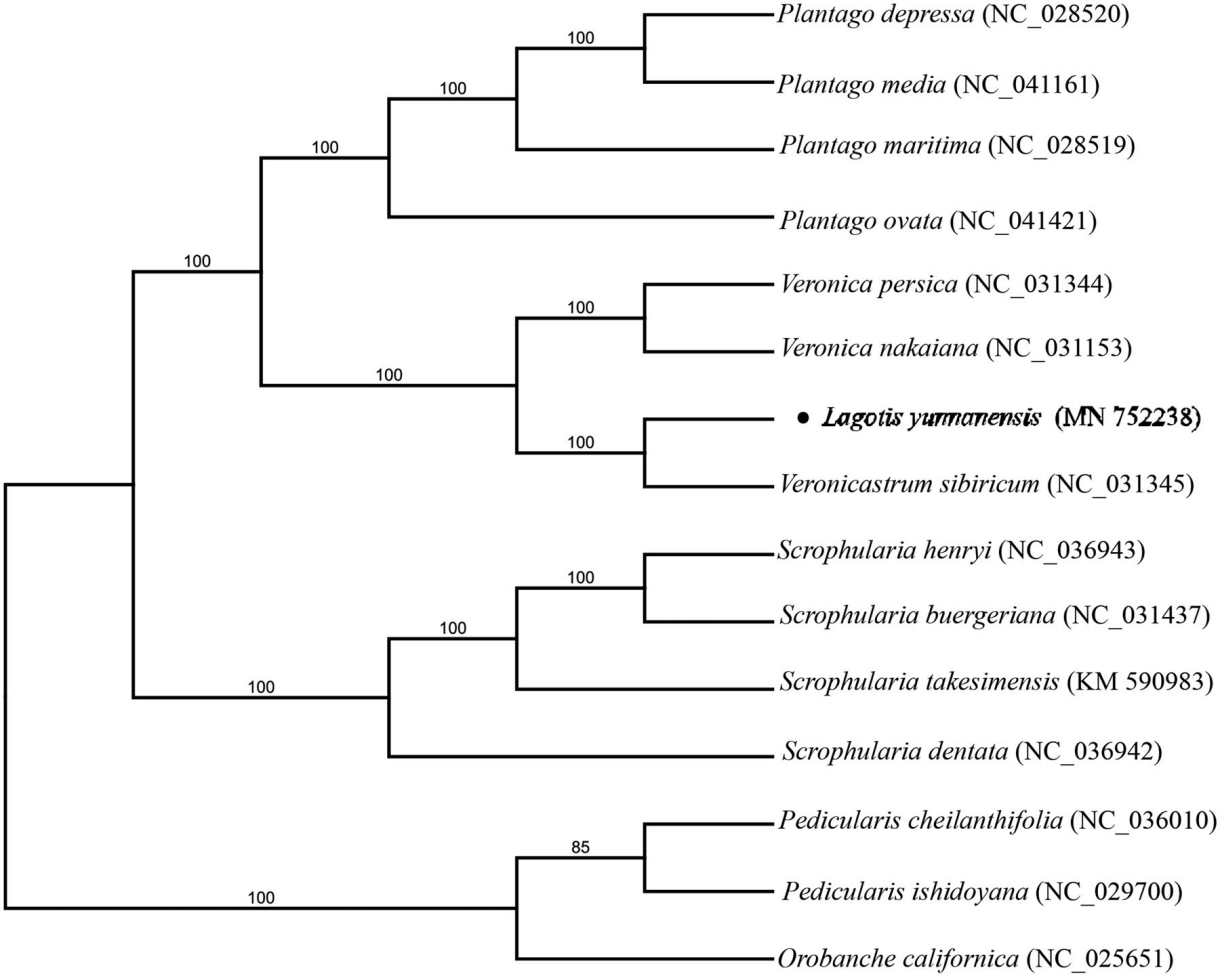
Maximum likelihood phylogenetic tree inferred from 15 chloroplast genomes. Bootstrap support values >50% are indicated next to the branches.
